# Assuring quality in microplastic monitoring: About the value of clean-air devices as essentials for verified data

**DOI:** 10.1038/s41598-017-05838-4

**Published:** 2017-07-14

**Authors:** Charlotte Wesch, Anna Maria Elert, Manuel Wörner, Ulrike Braun, Roland Klein, Martin Paulus

**Affiliations:** 1 Trier University, Department of Biogeography, Universitätsring 15, 54286 Trier, Germany; 2 Federal Institute for Material Research and Testing (BAM), Unter den Eichen 87, 12205 Berlin, Germany

## Abstract

Avoiding aerial microfibre contamination of environmental samples is essential for reliable analyses when it comes to the detection of ubiquitous microplastics. Almost all laboratories have contamination problems which are largely unavoidable without investments in clean-air devices. Therefore, our study supplies an approach to assess background microfibre contamination of samples in the laboratory under particle-free air conditions. We tested aerial contamination of samples indoor, in a mobile laboratory, within a laboratory fume hood and on a clean bench with particles filtration during the examining process of a fish. The used clean bench reduced aerial microfibre contamination in our laboratory by 96.5%. This highlights the value of suitable clean-air devices for valid microplastic pollution data. Our results indicate, that pollution levels by microfibres have been overestimated and actual pollution levels may be many times lower. Accordingly, such clean-air devices are recommended for microplastic laboratory applications in future research work to significantly lower error rates.

## Introduction

A high degree of quality assurance is a fundamental requirement to ensure global comparability of research results. However, well-established quality assurance schemes for microplastic (<5 mm)^1^ investigations, including all steps of sampling, processing and analysis, are missing. This may be attributed to a lack of quality-tested long term microplastic monitoring programmes^2^. Recently, efforts to ensure different quality criteria have been put forward but are still in their initial stages and not broadly applied^2^.

So far, existing microplastic records have highlighted the prevalence of microfibres in environmental samples^3, 4^. Fibres are the most common microplastic type monitored in sediments^5, 6^, surface seawaters^7^, aquatic biota^8, 9^ as well as atmospheric fallout^10^ and in indoor environments^11^. However, recent studies report about numerous sources of analytical errors like the misidentification of synthetic fibres with artificial cellulose or lignin fibres, due to insufficient spectroscopic measurements^12–14^. Moreover, microfibres in environmental specimens can be confounded with post-sampling contamination in the laboratory^15^. Without any prevention of potential background contamination a quantification of microfibres in environmental samples remains highly questionable.

Possible sources of background contamination with microfibres are numerous and can be caused by abrasions from synthetic clothing, inexpertly cleaned laboratory equipment, plastic tools used during processing, poorly sealed specimen or ambient air^16–18^. Some basic precautionary measures and methods to prevent aerial microfibre contamination have been recommended in the scientific literature: Clothing made of cotton should be worn^5, 12, 19, 20^, air circulation should be avoided^2^ and work spaces and tools need to be cleaned from any particle contamination^8^. The general elimination of microfibres from further analyses^21^ has also been postulated, considering all fibres to be artefacts of sample processing^22, 23^. However, this exclusion is no longer suitable to detect microfibres in environmental samples, as it distorts the factual results of contamination^15, 24, 25^. Thus, an analytical assessment of airborne contamination levels is needed considering the laboratory-specific pollution with microfibres^17^. Moist filter papers in Petri dishes exposed to the laboratory air could function as control blanks during an examination process^2^. To avoid fibre contamination in the laboratory, hermetic enclosure devices like plastic covers and a pyramid glove box were also tested to isolate all work spaces during processing^25^. Besides, textile fibre contamination was found to decrease, when processing was performed in a clean air flow cabinet^23^. Other studies used common laboratory clean-air devices like laminar flow cabinets^20, 26^ or fume hoods^16, 27^ during sample processing.

To assure the efficiency of laboratory clean-air devices in microplastic monitoring, we have tested and compared the airborne microfibre contamination of environmental samples in different experimental set ups: (1) indoor, (2) in a mobile laboratory, (3) within a laboratory fume hood and (4) on a clean bench with particles filtration. The object of this paper is to address the value of suitable clean-air devices for valid microplastic pollution data.

## Results

### Number of airborne fibres

**Figure 1:**
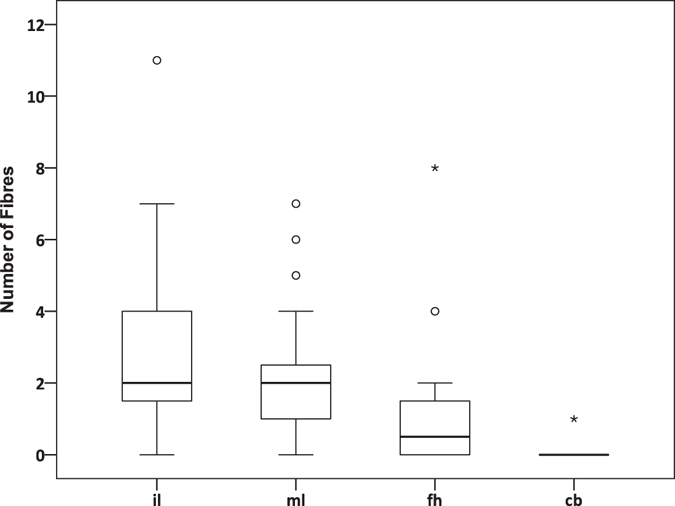
Number of aerial microfibres monitored in the samples. The boxplots show the number of airborne fibres detected in the samples belonging to the four different setups (il = indoor laboratory; ml = mobile laboratory; fh = laboratory fume hood; cb = clean bench with particle filtration). The boxes cover the 25th and 75th percentile, including the median (horizontal line). The whiskers are set to default representing ±1.5*IQR (interquartile range).

**Figure 2:**
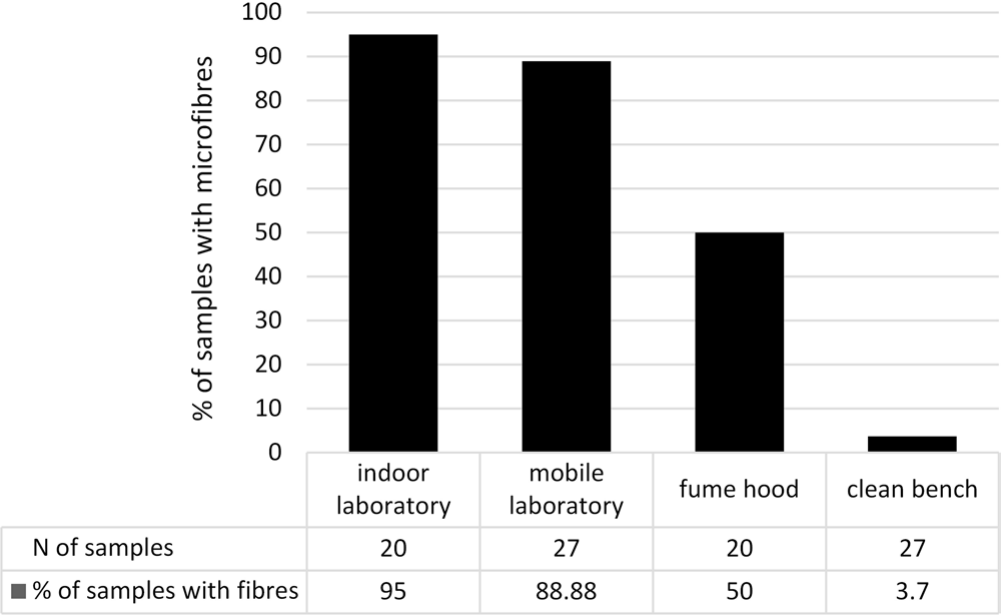
Percentage of samples with microfibre contamination. The figure shows the percentage of samples found with microfibre contamination indoor, in the mobile laboratory, the fume hood and on the clean bench with particle filtration. The number of samples exposed to the four different setups are represented as well. The entire list of samples exposed to the different setups can be found as Supplementary Tables S1 and S2.

**Figure 3:**
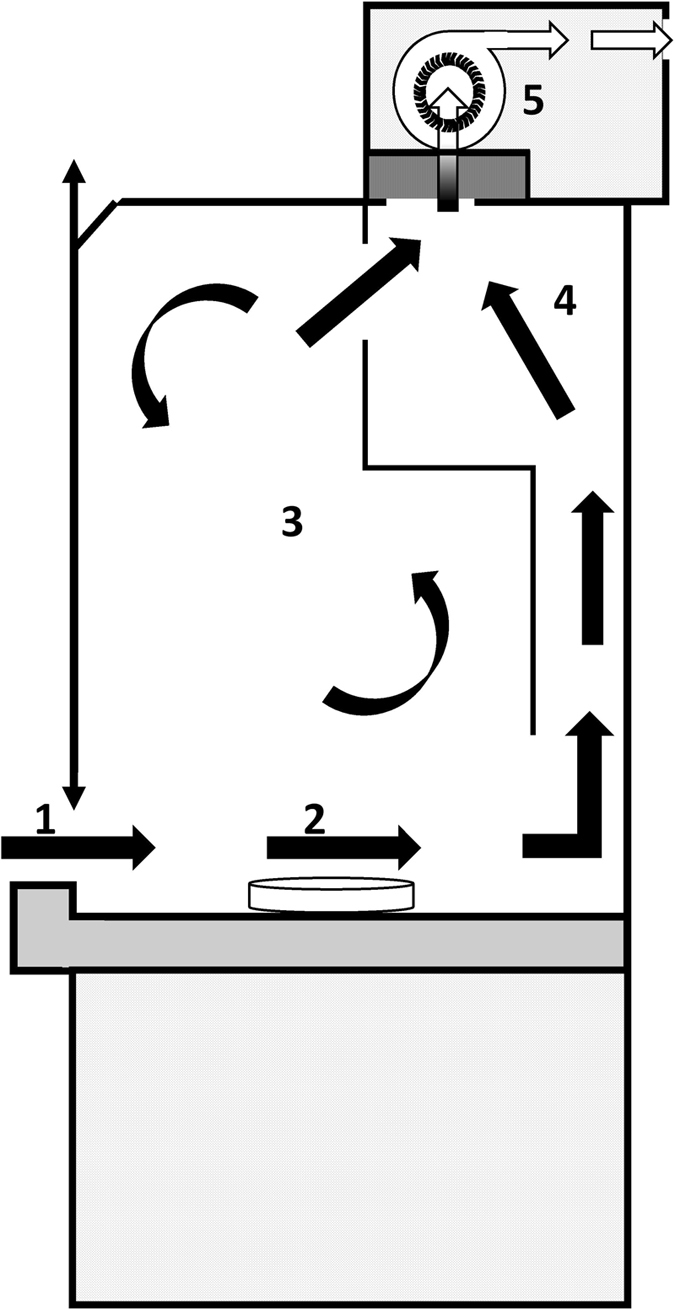
Image of a typical laboratory fume hood. A fume hood is a ventilated enclosure that provides protection of the operator and the environment from particularly hazardous substances like fumes, vapours, dusts and gases. During processing, ambient air flows into the fume hood through a vertically movable front sash (1). The unfiltered air flows across the work surface, over the sample (2) and circulates in the workspace (3). Air and all airborne contaminants are directed through an air exhaust duct (4) into the exhaust fan (5) and suctioned out of the fume hood.

**Figure 4:**
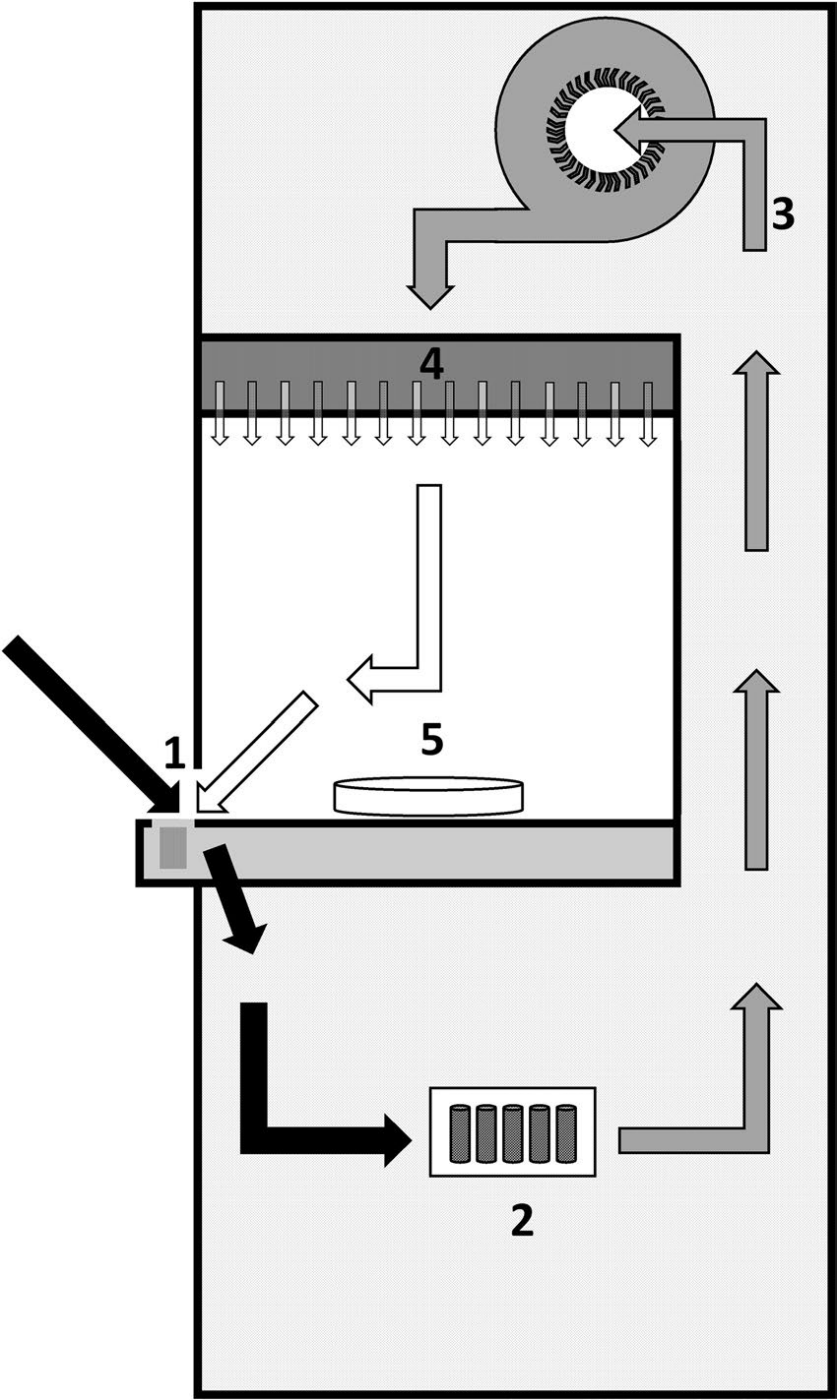
Image of a laboratory clean bench with particles- and activated carbon filtration. Clean benches provide a particle-filtered environment to protect samples from aerial contamination. Ambient air with floatable particles (black arrows) and filtered pure air (white arrows) are transported to suction slots (1) and passed through an activated carbon filter (2). Cleaned air is drawn by a radial fan (3) and pressed through a particle filter (Hosch-Filter) into the workspace (4). The filtered pure air flows gently (laminar flow) across the work surface over the sample (5) and towards the operator and then back into the suction slots.

Airborne fibres contamination levels from different setups (indoor laboratory, mobile laboratory, fume hood, clean bench with particles filtration) were tested and compared. A total of 138 fibrous appearances were counted in 54 of the overall 94 samples exposed to the four setups. Particle-shaped appearances were not found. All detected fibres were mostly black, blue or transparent. Figure 1 illustrates the number of fibres in the samples belonging to each setup. Microfibres abundance ranged from zero to 11 fibres per sample. The highest frequency of fibres was observed in the samples of the indoor setup, as nearly all samples contained fibres (Fig. 2). Likewise, 24 of the 27 samples of the mobile laboratory setup contained fibres. In the fume hood setup (Fig. 3), on the other hand, fibres were only detected in 10 of 20 samples. One single fibre was discovered in the samples of the clean bench setup (Fig. 4).

### Statistics

**Figure 5:**
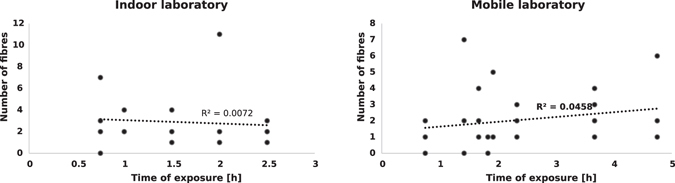
Linear regression analysis. Correlation between the number of fibres recovered on the filter papers and the time of exposure indoor (left) and in the mobile laboratory (right).

The sample size was tested to have an adequate power (>0.99). The differences in samples size was due to varying availability of fish samples for our experimental setups. The statistical tests showed that the amount of fibres collected in all samples exposed to the four different setups were significantly different (Kruskal-Wallis; df = 3; p = 0.000). The number of microfibres detected in the samples from the clean bench was significantly lower than the number of fibres collected either indoor (Wilcoxon Mann-Whitney U; Z = −6.089; p = 0.000), in the mobile laboratory (Z = −6.012; p = 0.000) or the fume hood (Z = −3.704; p = 0.000). No significant difference was found between the number of fibres recorded indoor and in the mobile laboratory (Table S3). The number of fibres indoor and the mobile laboratory was not significantly correlated to the time of exposure to the ambient air (Fig. 5 *;* linear regression analysis p > 0.05).

### Types of airborne fibres

**Figure 6:**
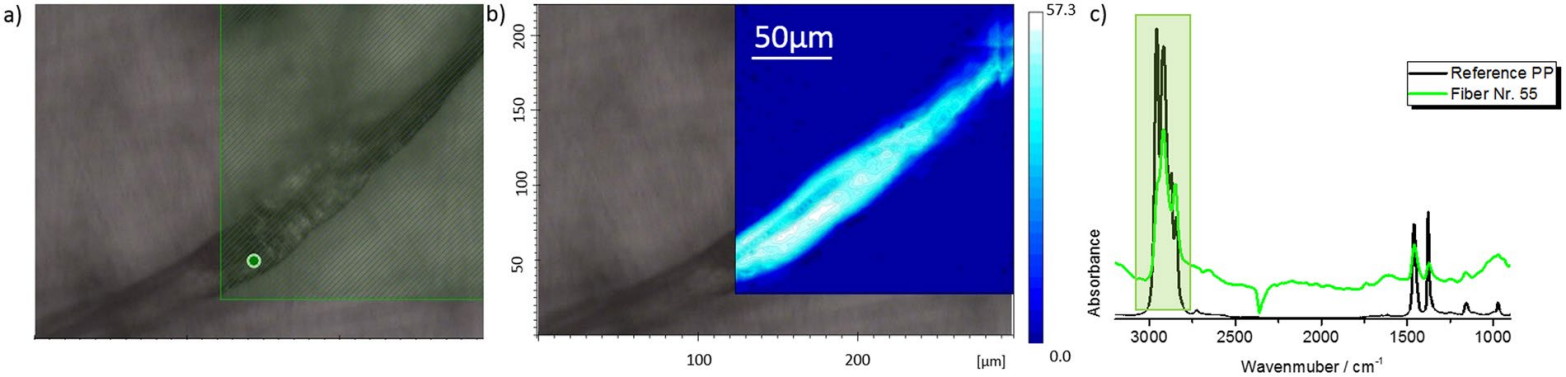
Optical image and spectra of an aerial fibre identified as polypropylene. The figure shows an optical image (**a**) with a corresponding FTIR chemical image integrated at 2800–3000 cm^−1^ (**b**). Image **c** is showing a fibre spectra (green) with its corresponding spectra (black; PP reference).

**Figure 7:**
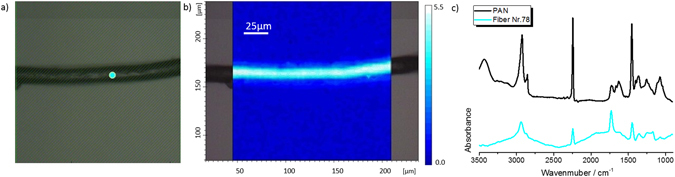
Optical image and spectra of an aerial fibre identified as polyacrylonitrile. The figure shows an optical image of a microfibre (**a**) with its corresponding FTIR chemical image (**b**), showing the PAN fibre at integration 2200–2300 cm^−1^. Image **c** shows the PAN reference spectrum (black) and the fibre’s spectrum (light blue).

**Figure 8:**
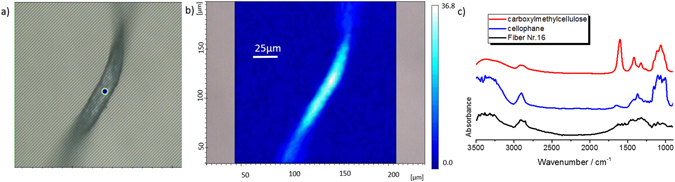
Optical image and spectra of an aerial fibre identified as cellophane. The figure shows an optical image of a microfibre (**a**) with corresponding FTIR chemical image (**b**), showing the studied fibre at integration 3000–2900 cm^−1^. CMC (red) and cellophane (blue) reference spectra with black corresponding to a microfibre (**c**).

Unfortunately, spectroscopic analyses could only be conducted for 13 of the 138 fibres, due to difficulties in handling the fibres during manual sample preparation and processing (e.g. loss of fibres or too small fibres), which is not unusual when dealing with tiny microfibres^28^. Six fibres were confirmed as microplastics, with the plastic polymer types identified as polypropylene (PP) (Fig. 6), polyacrylonitrile (PAN) (Fig. 7; Figure S1) and polyethylene terephthalate (PET) (Figure S2). But, six more fibres could be identified as of natural origin by the presence of melanin or cellulose derivatives, as e.g. contained in animal or human hair (Fig. 8). No assignments could be obtained for the thirteenth fibre. The size of the detected microfibres was between a few µm up to cm in length and from few µm up to 50 µm in thickness. Natural fibres however were, with up to 100 µm, generally thicker than the synthetic ones.

## Discussion

We highlight the need for greater attention to quality issues in order to identify and lower methodological errors in future microplastic investigations. Despite strict precautionary measures, the outcomes of our study indicate the high risk of aerial microfibre contamination of environmental samples during processing. Overall, common precautions like wearing 100% pure cotton lab coats and using non-plastic equipment were shown to be insufficient to ensure sample purity. Contamination of all but the clean bench setups were alarming. The single fibre found in the clean bench sample might have been released from laboratory equipment during processing. While the use of a laboratory fume hood reduces the contamination of specimens by almost 50%, it does not fully prevent it, as it is designed for the protection of the operator and ambient air from particularly hazardous substances, instead of protecting the sample from secondary contamination. Reason for the non-linear relationship between the number of fibres in the laboratory and the time of exposure, might be due to a variety of parameters. Movements in a room during processing (persons walking) for example might have an effect on the number of fibres settling down on the filter papers. Michele *et al*.^25^ already found no significant dependency between the amount of fibres in a sample and the time of exposure to the ambient air within their investigations. Procedural blank outcomes of recent studies do not indicate significant contamination levels in comparison to the levels found in environmental samples^10^. However, following our results, only standardised clean working conditions using suitable clean-air devices with particle filtration provide a sufficiently effective method to significantly reduce airborne microfibre contamination.

The results of our spectroscopic analyses show that the microfibres found in our samples were in equal parts of natural as well as of synthetic origin. Dris *et al*.^10^ also found this relation of natural to synthetic fibres in samples from the atmospheric fallout. The polymer types PP, PET and PAN detected in our study are often used in sanitary and personal care products^29^, as well as in the clothing and packaging industry^30^. Accordingly, their presence in samples could be a contamination signature of laboratory environments.

Notwithstanding the urgent call for quality assurance systems in microplastic analyses, many monitoring research studies still apply error-prone methodology. Likewise, for all steps of sampling, sample handling, processing and preparation prior to analysis, suitable methods to prevent contamination are missing in current monitoring protocols, guidances (cf. Recommendations by NOAA Marine Debris Program^19^ or Guidance on Monitoring of Marine Litter by MSFD TSGML^2^) and single research surveys. The MSFD TSGML^2^ recommended that background contamination should be considered as negligible, if it comprises less than 10% of the overall microplastic average across all samples. Our results suggest, that this target can be easily achieved and background contamination can even be reduced by 96.5% if using suitable clean-air devices. However, these results are limited to our specific laboratory and the used safety workbench and we do not aim to provide accurate evidence for overestimations of microfibres in the natural environment as a lot of scientific questions remain uncertain. We highly emphasise that more standardised research is needed and other variables should be taken into account as well.

Nevertheless, the predominance of microfibres in environmental samples might be linked to aerial fibres contamination. Consequently, suitable clean-air devices with particles filtration, designed for the protection of biological specimens should be taken into consideration by scientist for microplastic laboratory applications. We suggest this working procedure to be implemented in standard operating procedures for microplastic analysis of environmental samples.

## Methods

### Experimental setup and design

To verify the efficiency of clean-air devices in microplastic monitoring, we examined possible aerial contamination of environmental samples with microfibres in four different test setups. Airborne contamination was tested (1) indoor (laboratory without clean-air facilities), (2) in a mobile laboratory (fully housed within a vehicle without clean-air facilities and designed to function while stationary at a sampling site), (3) a laboratory fume hood and (4) on a clean bench with particles- and activated carbon filtration (microbiological safety workbench; Type ASW-UP, BLEYMEHL Reinraumtechnik). The used clean bench removes particulates from ambient air by a gentle laminar flow of the filtered air, which streams vertically through the entire work space, reliably transporting all floatable particles to suction slots.

### Sample preparation and preventing contamination

As samples, we used white filter papers (90 mm diameter) moistened with ultrapure water, imitating a moist surface of an aquatic environmental sample. Prior to the exposure, all filter papers were observed visually for any fibre contamination and prepared in cleaned glass Petri dishes on the clean bench to prevent any previous contamination. These Petri dishes were then exposed to the four different setups described above. In the laboratory, general precautionary measures were adopted like wearing clothing made of 100% cotton, avoiding air circulation in the laboratory and furthermore all work spaces as well as all used equipment was cleaned from any particle contamination with deionised water.

### Exposure and enumeration

Exposure included all steps of a routine sample processing (as applicable for fish dissection) following the standard operating procedures (SOP)^31, 32^ of the German Environmental Specimen Bank, providing high quality assurance standards for environmental monitoring. The period of exposure was analogous to the mean time needed for a standardised fish dissection and ranged between 0.75 and 4.75 hours. After processing, all samples were sealed temporarily in the Petri dishes. For counting all fibrous microplastics, the sealed samples were visually observed under a Greenough Stereo Microscope with Apochromatic Optics (Leica S8 APO). To ensure the accuracy of the counting, two independent observers enumerated them alternating for two minutes. After analysis, fibres which were easy to handle were transferred into small Eppendorf tubes for storage prior to spectroscopic analyses.

### Statistical data analysis

We initially determined the optimal sample size with an adequate power to detect the statistical significance (G*Power 3.1). Statistical data comparison was conducted using SPSS software (IBM SPSS Statistics 23.0). Data was tested for normal distribution using the Shapiro-Wilk test. As our data was not normally distributed, non-parametric tests were performed. The Kruskal-Wallis test was used to test the statistical significance of the differences of fibre contamination between the four different setups. Two tailed Wilcoxon Mann-Whitney U tests were applied to test for differences in quantity of fibres between the different setups. The relationship between the fibres recorded on the filter papers and the time of exposure was tested with linear regression analysis. The significance level (p) for the performed statistical tests was set at 0.05.

### Spectroscopic analysis

Fourier Transform infrared (FTIR) spectroscopy was used to chemically characterise microfibres, which were suggested to be of synthetic origin by their morphology. Measurements were carried out using a FTIR Vertex 70 spectrometer (Bruker) as beam source coupled with a Hyperion 3000 FTIR Microscope with a 15 x IR Objective (Bruker Corporation, Billerica, USA) and a 64 × 64 Focal Plane Array (FPA) detector. For analysis, fibres were uniformly spread on a KBr crystal and placed under a microscope equipped with a 15 × Objective (pixel resolution 2.7 µm). The measurements were performed in transmission mode in wavenumber range 3850–900 cm^−1^ using a spectra resolution of 16 cm^−1^. OPUS 7.2 Software was used to acquire and analyse data. Zero filling factor 2, Blackman Harris three term windows function were chosen. The background was measured with the same parameters against the KBr window. No transformation or post-processing of the spectra were conducted. To differentiate the fibres chemistry, the special integration was applied. For PP fibre CH-valence-stretching vibrations at 3000–2800 cm^−1^ and for PAN fibres specific vibration of CN absorption band at 2260 cm^−1^ were integrated.

### Data availability statement

There are no restrictions on the availability of materials or information. Supporting raw data and materials that cannot be included in the printed version of the manuscript for reasons of space are available as Supplementary Information file that will be freely accessible on nature.com upon publication.

## Electronic supplementary material

Supplementary information
