# Supplementary material for: Assuring quality in microplastic monitoring: About the value of clean-air devices as essentials for verified data
Source: Sci Rep. 2017 Jul 14;7:5424. doi: 10.1038/s41598-017-05838-4 (PMC5511265; doi:10.1038/s41598-017-05838-4)
Supplement: Supplementary file 1 — Supplementary information [file 41598_2017_5838_MOESM1_ESM.pdf]

Supplementary information

Assuring quality in microplastic monitoring:

About the value of clean-air devices as essentials for verified data

Authors: Charlotte Wesch [a,*], Anna Maria Elert [b], Manuel Wörner [a], Ulrike Braun [b], Roland Klein [a], Martin Paulus [a]

[a] Trier University, Department of Biogeography, Universitätsring 15, 54286 Trier, Germany

[b] Federal Institute for Material Research and Testing (BAM), Unter den Eichen 87, 12205 Berlin, Germany

* Corresponding author:
Trier University
Department of Biogeography
Universitätsring 15
54296Trier, Germany
E-mail address: wesch@uni-trier.de

Table S1. Raw data of airborne microfibre contamination monitored in the procedural blanks. The table shows the samples belonging to the different setups, time of exposure, the number of fibres detected and the polymer types identified.

ID	setup	exposure time [h]	n of aerial fibres detected	polymer types
3	mobile lab	4.75	2	
4	mobile lab	4.75	1	
5	mobile lab	4.75	2	
6	mobile lab	4.75	6	
9	mobile lab	2.33	3	
10	mobile lab	2.33	1	
11	mobile lab	2.33	2	
12	mobile lab	2.33	1	
15	mobile lab	3.67	4	
16	mobile lab	3.67	3	cellulose/ cellophane
17	mobile lab	3.67	2	
18	mobile lab	3.67	1	presence of melanin
33	mobile lab	1.83	0	
34	mobile lab	1.83	1	
35	mobile lab	1.83	1	
43	mobile lab	1.67	2	
44	mobile lab	1.67	1	
45	mobile lab	1.67	4	
48	mobile lab	1.42	7	
49	mobile lab	1.42	2	presence of melanin
50	mobile lab	1.42	0	
53	mobile lab	1.92	5	could not be identified
54	mobile lab	1.92	1	polyacrylonitrile
55	mobile lab	1.92	1	polypropylene
58	mobile lab	0.75	2	polypropylene
59	mobile lab	0.75	1	polyethylene terephthalate
60	mobile lab	0.75	0	
21	indoor lab	2.5	1	cellulose/ cellophane
22	indoor lab	2.5	2	
23	indoor lab	2.5	3	
24	indoor lab	2.5	1	
27	indoor lab	1.5	1	
28	indoor lab	1.5	4	
29	indoor lab	1.5	4	
30	indoor lab	1.5	2	
38	indoor lab	1	4	
39	indoor lab	1	2	
40	indoor lab	1	4	
63	indoor lab	0.75	3	presence of melanin

ID	setup	exposure time [h]	n of aerial fibres detected	polymer types
64	indoor lab	0.75	2	
65	indoor lab	0.75	2	
66	indoor lab	0.75	7	presence of melanin
67	indoor lab	0.75	0	
77	indoor lab	2	2	
78	indoor lab	2	11	polyacrylonitrile
79	indoor lab	2	1	polyacrylonitrile
80	indoor lab	2	2	
68	fume hood	1	2	
69	fume hood	1	1	
70	fume hood	1	4	
71	fume hood	1	1	
72	fume hood	1	0	
73	fume hood	1	0	
81	fume hood	1	1	
82	fume hood	1	8	
83	fume hood	1	2	
84	fume hood	1	0	
85	fume hood	1	4	
86	fume hood	1	1	
87	fume hood	1	0	
88	fume hood	1	0	
89	fume hood	1	1	
90	fume hood	1	0	
91	fume hood	1	0	
92	fume hood	1	0	
93	fume hood	1	0	
94	fume hood	1	0	
1	clean bench	4.75	0	
2	clean bench	4.75	0	
7	clean bench	2.33	0	
8	clean bench	2.33	0	
13	clean bench	3.67	0	
14	clean bench	3.67	0	
19	clean bench	2.5	0	
20	clean bench	2.5	0	
25	clean bench	1.83	0	
26	clean bench	1.83	0	
31	clean bench	1.83	0	
32	clean bench	1.83	0	
36	clean bench	1	0	
37	clean bench	1	0	

ID	setup	exposure time [h]	n of aerial fibres detected	polymer types
41	clean bench	1.67	1	
42	clean bench	1.67	0	
46	clean bench	1.42	0	
47	clean bench	1.42	0	
51	clean bench	1.92	0	
52	clean bench	1.92	0	
56	clean bench	0.75	0	
57	clean bench	0.75	0	
61	clean bench	0.5	0	
62	clean bench	0.5	0	
74	clean bench	2	0	
75	clean bench	2	0	
76	clean bench	2	0	

Table S2. Raw data of aerial fibres contamination levels from the four different setups. The table shows the number of samples belonging to each setup as well as the number and percentage of samples contaminated with fibres.

setup	n of samples	n of samples with fibres	% of samples with fibres
indoor laboratory	20	19	95
mobile laboratory	27	24	88,88
fume hood	20	10	50
clean bench	27	1	3,7

Table S3. Differences in quantity of fibres between the different setups. The results of the two tailed Wilcoxon Mann-Whitney U tests are presented. The significance level (p) for the performed statistical test was set at 0.05.

	indoor laboratory	mobile laboratory	fume hood	clean bench
**indoor laboratory**	###	0.141	0.002	0.000
**mobile laboratory**	0.141	###	0.016	0.000
**fume hood**	0.002	0.016	###	0.000
**clean bench**	0.000	0.000	0.000	###

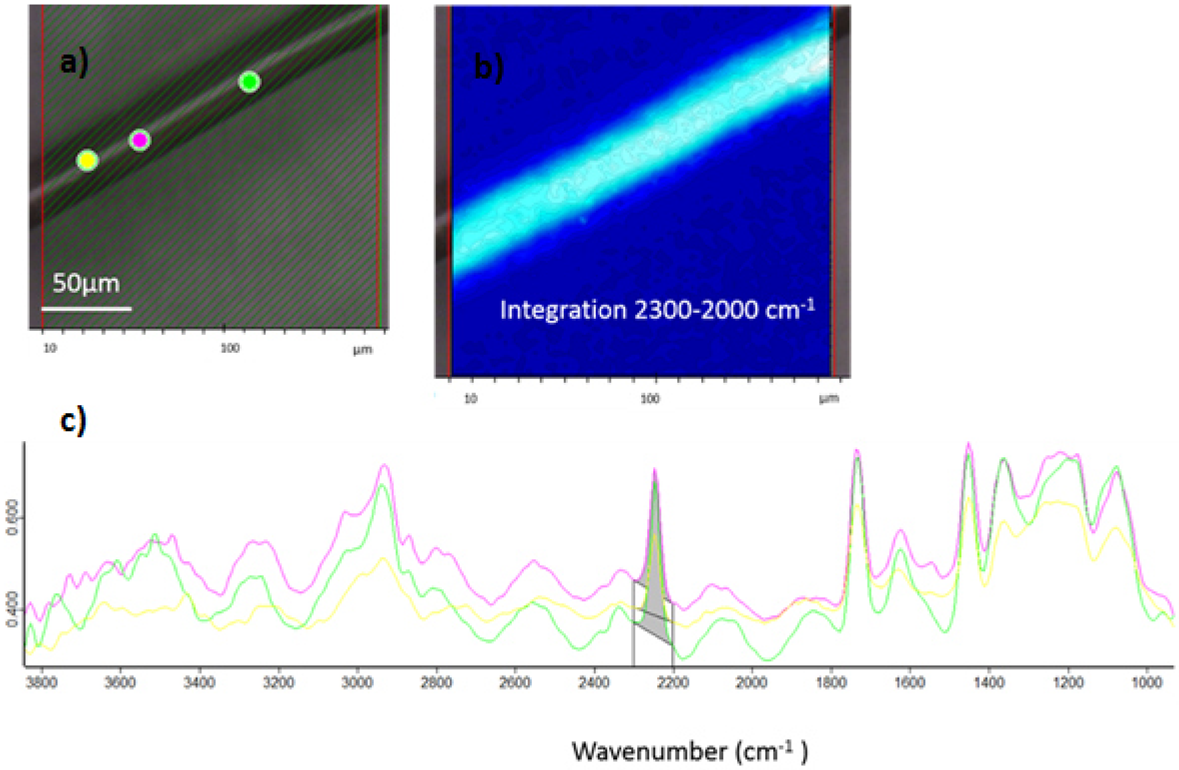

Figure S1: Optical image and spectra of an aerial fibre identified as polyacrylonitrile. An optical image of fibre No 54 (a) with the corresponding FTIR chemical image integrated at 2300-2200cm^{-1} (b) and the raw spectra (c).

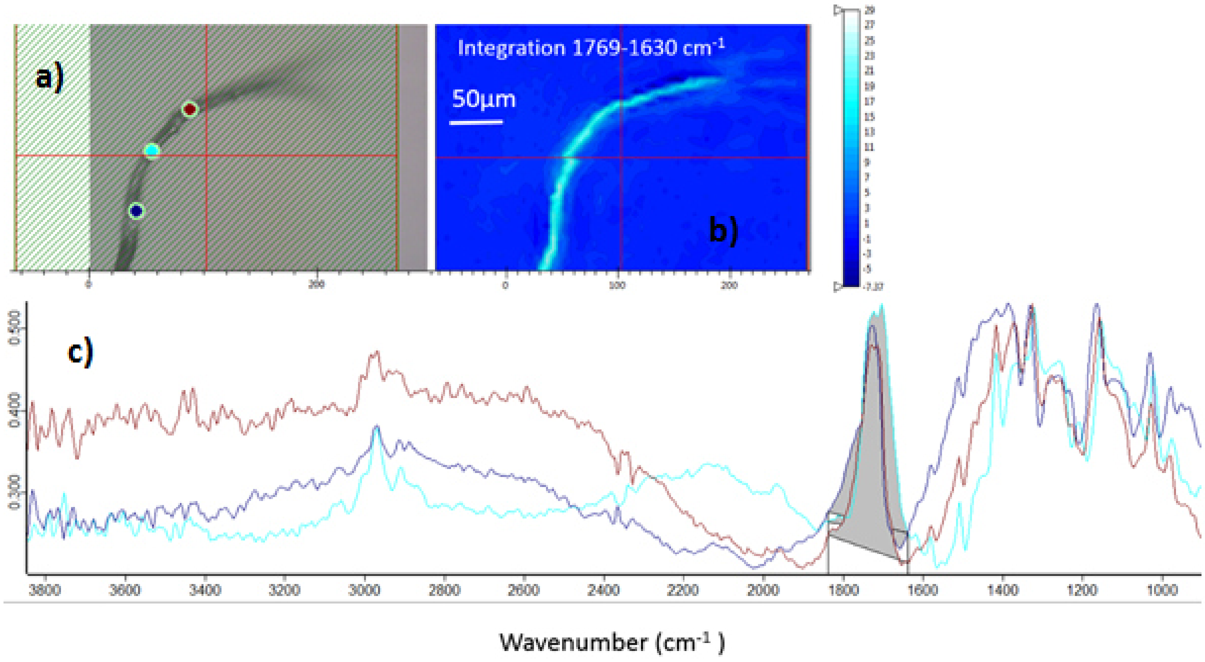

Figure S2. Optical image and spectra of fibre No 59 identified as polyethylene terephthalate. An optical image of fibre No 59 (a) with corresponding FTIR chemical image integrated at 1769-1630 cm^{-1} (b) and raw spectra (c).
